# Central adiposity is associated with poorer functional capacity in women with knee osteoarthritis: a cross-sectional study with exploratory adjustment for pain intensity and radiographic severity

**DOI:** 10.1007/s00296-026-06245-7

**Published:** 2026-07-14

**Authors:** Josiel Gomes Ribeiro, Gabriela Martins Alvarez, Giorgio Silvano Ferreira Poletto, Marcelo Peduzzi de Castro, Walter Herzog, Heiliane de Brito Fontana

**Affiliations:** Musculoskeletal Biomechanics Research Group, Federal University of Santa Catarina, BSiM-UFSC, University Campus Reitor João David Ferreira Lima, s/nº, Trindade, Florianópolis, SC 880359727 Brazil

**Keywords:** Osteoarthritis, Knee, Waist circumference, Anthropometry, Ultrasonography, Sarcopenia, Physical functional performance

## Abstract

To investigate the associations of waist circumference (WC) and calf circumference (CC) with functional performance in women with symptomatic radiographic knee osteoarthritis (OA), and to explore whether lower-leg muscle quality influences the relationship between CC and functional outcomes. This exploratory cross-sectional study included 28 women with symptomatic radiographic knee OA (Kellgren–Lawrence grades II–IV). WC and CC were measured using standardized anthropometric procedures andCC was analyzed as both raw and after correction for subcutaneous fat-thickness. Functional performance was evaluated using the 6-minute walk test (6MWT) and the 30-second chair stand test (30sCST). Lower-leg muscle quality was estimated using ultrasound echo intensity. Associations between anthropometric measures and functional outcomes were examined using Spearman correlation coefficients. Associations identified in correlation analyses were further explored using regression models to examine the potential influence of lower-leg muscle quality on the associations involving CC and to examine influence of pain intensity and radiographic severity on the associations of WC with functional performance. Greater WC was associated with poorer performance in both the 6MWT (ρ = −0.706, p = 0.001) and the 30sCST (ρ = −0.648, p = 0.001). Associations between CC and functional performance were weaker and less consistent. In the most affected limb, CC was not associated with 6MWT distance and showed a borderline association with 30sCST performance (ρ = −0.360, p = 0.060). In the contralateral limb, greater CC was inversely associated with 30sCST performance before (ρ = −0.482, p = 0.009) and after correction for subcutaneous fat thickness (ρ = −0.452, p = 0.016). These associations were no longer statistically significant after adjustment for lower-leg muscle quality. In the primary regression models, WC remained as the only significant predictor and explained 53% of the variance in 6MWT distance and 37% of the variance in 30sCST performance. Forced adjustment for pain intensity and radiographic severity produced only minimal changes in the WC coefficients. In women with symptomatic radiographic knee OA, greater WC was consistently associated with poorer functional performance, supporting its potential value as a simple anthropometric marker of functional vulnerability. CC did not behave as an indicator of better functional capacity; when associations were observed, they were inverse and appeared to be influenced by muscle quality, suggesting that CC alone may not adequately reflect functional muscle mass in this population. Larger longitudinal studies are needed to confirm these findings and clarify their clinical utility.

**Supplementary Information:**

The online version contains supplementary material available at 10.1007/s00296-026-06245-7.

## Introduction

Osteoarthritis (OA) is a chronic joint disorder characterized by dysregulated repair processes influenced by mechanical loading, inflammation, and metabolic factors. Progressive failure of the synovial joint involves changes in articular cartilage, subchondral bone, ligaments, capsule, and synovium, resulting in pain and functional limitation [1, 2]. Knee OA is the most prevalent presentation and a leading cause of disability in older adults, with a particularly high burden among women [2, 3].

Among modifiable factors involved in the development and progression of knee OA, obesity and metabolic syndrome have received increasing attention due to their high prevalence and potential responsiveness to conservative management [4]. Visceral adiposity may influence OA symptoms and progression not only through increased joint loading, but also through systemic low-grade inflammation and adipokine-mediated pathways that can affect joint tissues and pain sensitivity [5–8]. In parallel, excess adiposity is associated with unfavorable skeletal muscle adaptations, including impaired muscle composition and function, which may further impair mobility in people with OA. These joint and muscle pathways are likely interrelated, and emerging evidence supports a more central role for skeletal muscle in OA pathophysiology and in the expression of disability [9–11].

This integrated perspective has motivated interest in body-composition phenotypes that combine adiposity and low muscle mass or function, commonly referred to as sarcopenic obesity (SO). SO is hypothesized to exacerbate functional decline through the convergence of central adiposity, systemic metabolic inflammation, and diminished muscle capacity [9, 12, 13]. In addition, associations between central obesity and symptomatic OA have also been reported in non-weight-bearing joints such as the hand, supporting the concept that adiposity-related mechanisms may contribute to OA beyond local biomechanical loading [14]. Despite these mechanistic links, evidence remains limited on whether simple clinical measures reflecting central adiposity and peripheral muscle mass can discriminate functional capacity in women with knee OA.

Waist circumference (WC) is a widely used, low-cost, practical anthropometric indicator of central adiposity in cardiometabolic risk assessment and a main predictor of comorbidities in metabolic syndrome [15, 16]. Yet, despite its clinical simplicity, WC is not routinely incorporated into OA assessment, and its relationship with performance-based functional capacity in individuals with established symptomatic OA remains insufficiently characterized [17]. Calf circumference (CC), another simple anthropometric measure, has been proposed as a proxy for lower-limb muscle mass and as a potential indicator of functional status [18, 19]. However, little is known regarding its utility in the context of OA. In individuals with higher adiposity, CC may not reflect muscle mass or muscle capacity. Subcutaneous fat and altered muscle quality, particularly intramuscular fat infiltration and fibrosis, have been consistently reported in knee OA and may decouple circumference-based measures of muscle mass from functional capability [10, 11, 20].

Functional limitations, such as difficulty walking and rising from a chair are key determinants of quality of life and are often weakly related to radiographic severity [21, 22]. Accordingly, current guidelines recommend prioritizing function-centered assessments rather than relying solely on joint structural measures [23]. In this context, establishing whether simple, widely accessible anthropometric measures can help identify functional impairment would offer practical value for clinical screening and for targeting conservative interventions.

Therefore, we investigated the associations of WC and CC with functional capacity in women with symptomatic, radiographic knee OA. We further evaluated whether the relationship between CC and functional performance is influenced by subcutaneous fat thickness and lower-leg muscle quality. We hypothesized that greater WC is associated with poorer functional performance, and that lower CC would be associated with poorer function once the potential confounding effects of adiposity and muscle quality were considered.

## Materials and methods

### Study design and ethical approval

This cross-sectional analysis used data from the project “Hemodynamic Muscle Response in Individuals with Osteoarthritis: Relationship Between Contraction-Induced Reactive Hyperemia, Pain, Strength, and Muscle Quality – HIPEROA”, conducted by the Musculoskeletal Biomechanics Research Group at the Federal University of Santa Catarina. The broader project investigates muscle-related determinants of functional capacity in women with knee osteoarthritis (OA), including assessments of microvascular function, muscle quality, strength, and anthropometric characteristics. The study was approved by the Human Research Ethics Committee of the Federal University of Santa Catarina (CAAE: 42610820.8.0000.0121; Opinion No. 5189446. Approval date: December 29, 2021) and conducted in accordance with the Declaration of Helsinki. All participants provided written informed consent prior to participation. This study was reported according to the STROBE (Strengthening the Reporting of Observational Studies in Epidemiology) guidelines for cross-sectional studies, and the completed checklist is available as supplementary material.

### Participants

Sample size estimation was performed a priori using G*Power software (version 3.1.9.7; Heinrich Heine University Düsseldorf, Germany). An exact test for a two-tailed bivariate normal correlation model was selected. Assuming a moderate expected effect size (*r* = 0.50), α = 0.05, and statistical power of 80%, the estimated minimum sample size was 29 participants.

Women aged 50–75 years visiting our orthopedic clinic for OA-related symptoms between 2020 and 2021 were screened for eligibility. Potentially eligible participants were identified from clinic screening records. Inclusion criteria were: (i) female sex; (ii) diagnosis of knee OA according to the American College of Rheumatology clinical criteria; and (iii) disease severity classification II or higher according to the Kellgren–Lawrence (KL) radiographic scale. Exclusion criteria were: (i) radiographic KL grade II–IV without clinical symptoms (i.e., asymptomatic on WOMAC pain, stiffness, or function domains); (ii) subcutaneous fat thickness greater than 2 cm at the medial gastrocnemius of the most affected limb [because of the limitations of near-infrared spectroscopy which was used in broader HIPEROA project to assess gastrocnemius muscle hyperemic response]; (iii) inability to walk; (iv) presence of an infectious process; (v) surgery or trauma within the 6 months prior to data collection; (vi) systemic conditions that could affect vascular or exercise safety (e.g. thrombosis, uncontrolled hypertension, cancer, or any condition preventing physical activity based on medical advice); (vii) corticosteroid injection within the preceding 3 months; (viii) presence of unstable angina or acute myocardial infarction within the month preceding data collection.

A total of 116 women identified from clinic screening records were contacted by telephone and screened for eligibility and availability. This was a non-probabilistic convenience sample. Detailed reasons for non-inclusion among all contacted individuals were not systematically recorded; however, most did not meet the eligibility criteria or were unavailable to participate during the data collection period. Thirty women attended the in-person evaluation sessions. Of these, one participant was unable to complete the assessments due to logistical reasons and another due to health-related reasons. Consequently, 28 women were included in the final analysis. No subjects evaluated exceeded the calf subcutaneous fat thickness criterion ≤ 2 cm,

### Anthropometric assessments

Body mass and height were measured using a digital scale (G-Tech; 0.1-kg resolution) and a stadiometer (1-mm resolution), and body mass index (BMI) was calculated as kg/m². Waist circumference (WC) was measured with the participant standing at the end of a normal expiration using a non-elastic measuring tape (1.5 m length; 1-mm graduation). The tape was positioned at the midpoint between the iliac crest and the lower margin of the last palpable rib in the mid-axillary line. Three consecutive measurements were obtained and averaged. Metabolic risk was classified according to WHO cutoffs for women: WC ≥ 80 cm indicating increased risk and ≥ 88 cm indicating substantially increased risk [24].

Calf circumference (CC) was measured as the largest circumference perpendicular to the longitudinal calf axis, with the participant standing and feet 20 cm apart. Three measurements were obtained for each limb and averaged. CC was analyzed both as the raw perimeter and after correction for subcutaneous fat thickness. For the correction, the model proposed by Stewart et al. [25] was adapted and calf diameter was estimated by dividing the measured perimeter by π. Corrected diameter was calculated by subtracting subcutaneous fat thickness observed in the gastrocnemius echogram. Corrected circumference was subsequently derived by multiplying the corrected diameter by π. All values are reported in centimeters.

### Radiographic severity, muscle quality and pain

Radiographic OA severity was assessed using the Kellgren–Lawrence (KL) radiographic scale. Knee radiographs were graded by an experienced clinician and recorded as an ordinal variable (II–IV).

Lower-leg muscle echogenicity (muscle quality) and subcutaneous fat thickness were assessed using B-mode ultrasound (LOGIQ S7 Expert; General Electric, USA). For the tibialis anterior, the probe was positioned at the proximal third of the distance between the apex of the patella and the lateral malleolus. For the medial gastrocnemius, images were obtained at 30% of leg length (distance from the popliteal fossa to the medial malleolus). The transducer was oriented perpendicular to the skin and aligned longitudinally with the muscle belly. Imaging parameters were standardized, including focus adjusted to the region of interest, following recommendations for minimizing depth-related bias [26]. Two sonograms per muscle were obtained for each leg. Subcutaneous fat thickness in the evaluated region was also quantified.

Sonograms were exported in DICOM format and analyzed using ImageJ (National Institutes of Health, USA). A rectangular region of interest (ROI; 1 cm height × 4 cm width) was positioned immediately below the superficial aponeurosis. Mean grayscale values (arbitrary units; range 0–255) were extracted within each ROI. Subcutaneous fat thickness was measured at three points per image and averaged. Echo intensity estimates of muscle quality were corrected for subcutaneous fat thickness using the equation described by Neto Müller et al. [26] for adjusted-focus acquisitions. Lower-leg muscle quality was computed as the mean corrected echo intensity from the tibialis anterior and medial gastrocnemius. All ultrasound image acquisitions and analyses were performed by a single trained evaluator following a standardized protocol developed within the broader research project. Participant positioning, probe placement, ultrasound settings (frequency, gain, depth, and focal adjustments), and image acquisition procedures were standardized across all assessments to minimize measurement variability. Echo intensity analysis and ROI placement procedures followed the methodological framework described by Alvarez (2024) [27]. Previous reliability analyses of the ultrasound protocol conducted in our laboratory demonstrated excellent reproducibility. Inter-rater reliability for a given sonogram showed intraclass correlation coefficients (ICC [1, 2]) greater than 0.934 for all measurements and conditions. Test-retest reliability yielded ICC (2,3) values greater than 0.84 (*p* < 0.001) for echo intensity and greater than 0.953 (*p* < 0.001) for adipose tissue thickness across all conditions [26]. In the present study, all ultrasound image analyses were performed by a single evaluator, who was blinded to participants’ clinical, anthropometric, and functional data during image analysis. Reliability of ROI placement was not reassessed in the current sample, as previously published reliability data from our laboratory were used to support the adopted methodology. Pain intensity was analyzed for the most affected side using the visual analogue scale (VAS). Measurements were conducted prior to functional tests.

### Functional performance

Functional capacity was assessed using the 30-second Chair Stand Test (30sCST) and the Six-Minute Walk Test (6MWT), consistent with recommendations for performance-based outcomes in OA [23].

For the 30sCST, participants sat on a 43-cm chair without backrest or armrests and performed as many full sit-to-stand cycles as possible within 30 s, with arms crossed over the chest and feet flat on the floor. Repetitions were counted from video recordings.

For the 6MWT, participants walked indoors on a 2.1 m × 9.5 m wooden course in the Biomechanics Laboratory of the Federal University of Santa Catarina. Two cones positioned 7 m apart defined the turning points. All participants were assessed under identical testing conditions using the same standardized protocol, ensuring internal consistency across assessments. Participants were instructed to walk as fast as possible for six minutes, with the option to rest if needed. Standardized verbal encouragement was provided every minute, including a reminder during the final minute. Total walking distance was calculated from the number of completed laps (14 m per lap) plus the final partial distance according to the participant’s finishing position.

### Statistical analysis

Normality was assessed using the Shapiro–Wilk test. Considering the exploratory nature of the sample size in this study, associations between anthropometric measures (waist circumference [WC] and calf circumference [CC]) and functional outcomes (6MWT distance and 30sCST repetitions) were first examined using Spearman correlation coefficients. CC analyses were conducted using both raw and fat-corrected values.

Associations involving CC that were statistically significant in the correlation analyses were subsequently examined using linear regression models adjusted for lower-limb muscle quality, defined as the mean corrected echo intensity of the tibialis anterior and medial gastrocnemius.

For the WC analyses, age, body mass index (BMI), VAS pain score, KL grade, and raw and corrected limb circumference measures were evaluated as candidate predictors during exploratory stepwise model building. Variable entry and removal criteria were set at *p* < 0.05 and *p* > 0.10, respectively.

As a post hoc sensitivity analysis, forced-entry multivariable linear regression models were performed to examine whether the association between WC and functional performance was altered after adjustment for pain intensity and radiographic severity. WC, VAS pain score, and KL grade were entered simultaneously into each model, irrespective of statistical significance. BMI was not forced into these sensitivity models because of its conceptual and statistical overlap with WC and the limited sample size. Consequently, these analyses were not intended to determine whether the WC associations were independent of BMI The WC coefficients obtained from these models were compared with those from the primary univariable models. All analyses were performed using JASP version 0.18.3, with statistical significance set at *p* < 0.05. No missing data was observed for the variables included in the analyses; therefore, no imputation procedures were required.

## Results

Twenty-eight women with symptomatic radiographic knee OA participated in the study (age: 61 ± 7 years; BMI = 28 ± 4 kg/m²). Radiographic severity was KL grade II in 64% of participants and KL grades III–IV in 36%. Mean pain intensity was 5 ± 2 points on the VAS. Based on WC, 23% of participants were classified as having low metabolic risk, 40% having increased risk, and 37% as having substantially increased risk.

### Associations between waist circumference and functional capacity

Greater waist circumference (WC) was associated with poorer functional performance in both the 6MWT (ρ = −0.706, 95% CI: −0.854 to − 0.452, *p* = 0.001) and the 30sCST (ρ = −0.648, 95% CI: −0.822 to − 0.362, *p* = 0.001) (Table 1).

**Table 1:** Mean ± SD of Waist Circumference (WC) and Calf Circumferences (CC) and their relationship with the number of repetitions in the STS30 and the distance reached in the 6MWT

Anthropometric Variables	Mean (SD)	Spearman Correlation rho (95%CI)				
		6MWT(m)		ρ	30sCST (reps)	ρ
WC (cm)	87 ± 11.2	−0.706 (−0.854; −0.452)		0.001	−0.648 (−0.822; −0.362)	0.001
CC (most affected, cm)	38 ± 3.4	−0.202 (−0.535; −0.185)		0.303	−0.360 (−0.646; 0.015)	0.060
CC (most affected, corrected, cm)	35 ± 2.7	−0.273 (−0.586; 0.112)		0.160	−0.355 (−0.643; 0.020)	0.063
CC (contralateral, cm)	38 ± 3.4	−0.314 (−0.615; −0.067)		0.104	−0.482 (−0.725; −0.133)	0.009
CC (contralateral, corrected cm)	35 ± 2.7	−0.372 (−0.654; 0.001)		0.051	−0.452 (−0.706; −0.094)	0.016

WC = waist circumference; CC = calf circumference; 30sCST = 30-Second Sit-to-Stand Test; 6MWT = 6-Minute Walk Test

### Associations between calf circumference and functional capacity

Associations between calf circumference (CC) and functional performance were generally weaker and less consistent than those observed for WC (Table 1). For the most affected limb, CC was not significantly associated with 6MWT distance (ρ = −0.202, *p* = 0.303) or with 30sCST repetitions (ρ = −0.360, *p* = 0.060). After correction for subcutaneous fat thickness, associations remained non-significant for both 6MWT (ρ = −0.273, *p* = 0.160) and 30sCST (ρ = −0.355, *p* = 0.063).

For the contralateral limb, CC demonstrated moderate inverse associations with functional performance, particularly with 30sCST performance (ρ = −0.482, *p* = 0.009). Similar findings were observed after correction for subcutaneous fat thickness (ρ = −0.452, *p* = 0.016). Associations with 6MWT distance were weaker and of borderline statistical significance after correction (ρ = −0.372, *p* = 0.051).

Lower-leg muscle quality, estimated by ultrasound echo intensity, averaged 171.1 ± 18.3 a.u. in the most affected limb and 176.7 ± 15.0 a.u. in the contralateral limb, with higher values indicating poorer muscle quality. After adjustment for muscle quality, calf circumference was no longer associated with functional performance outcomes (*p* > 0.10), suggesting that the inverse associations observed in unadjusted analyses may primarily reflect tissue composition, including adiposity and impaired muscle quality, rather than muscle size per se.

### Radiographic severity, pain and functional capacity

Radiographic severity and pain intensity both showed weak, non-significant inverse associations with functional performance. For radiographic severity, the correlation coefficients were ρ = −0.28 (*p* = 0.20) for 6MWT distance and ρ = −0.27 (*p* = 0.21) for 30sCST performance. For pain intensity, the corresponding coefficients were ρ = −0.31 (*p* = 0.10) and ρ = −0.28 (*p* = 0.15), respectively.

**Fig. 1:**
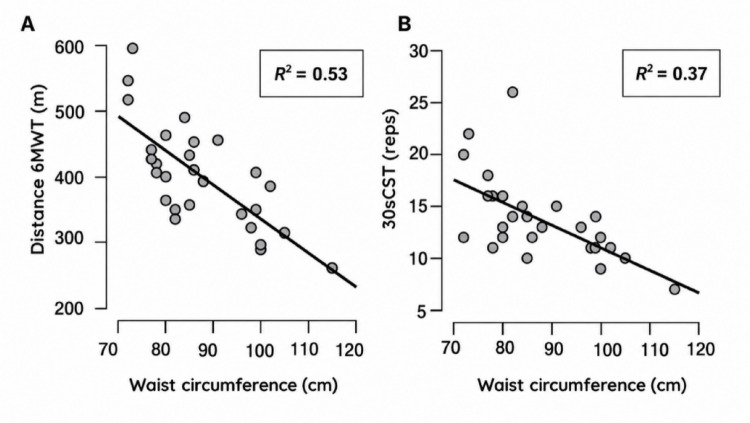
Associations between waist circumference and functional performance in women with symptomatic radiographic knee osteoarthritis. Scatterplots show the associations of waist circumference with **A** six-minute walk test distance and **B** 30-second chair stand test repetitions. Waist circumference was the sole predictor in each model (see text for details). Model coefficients, confidence intervals and explained variance are presented in Table 2

Age, BMI, pain intensity, KL grade, and raw and corrected limb circumference measures were evaluated as candidate variables during exploratory stepwise model building. None met the retention criteria, leaving WC as the sole predictor in the final models. These primary univariable models indicated that WC was associated with both 6MWT distance and 30sCST performance, explaining 53% and 37% of the variance in these outcomes, respectively (Table 2; Fig. 1). For every 1-cm increase in WC, 6MWT distance decreased by an estimated 5.33 m (B = − 5.33, 95% CI − 7.69 to − 2.97, *p* < 0.001), while 30sCST performance decreased by an estimated 0.22 repetitions (B = − 0.22, 95% CI − 0.33 to − 0.10, *p* < 0.001).

**Table 2:** Associations of waist circumference with functional performance outcomes

Outcome	Predictor	B	SE	β	95% CI	*P*		*R* ^2^	Adjusted *R*²
6MWT	WC	−5.33	1.13	−0.73	−7.69 to −2.97	< 0.001		0.53	0.50
30sCST	WC	−0.22	0.06	−0.61	−0.33 to −0.10	< 0.001		0.37	0.35

Exploratory model building was initially performed using a stepwise procedure. Age, body mass index, VAS pain score, KL grade, affected-limb calf circumference, corrected affected-limb calf circumference, and corrected contralateral-limb calf circumference were evaluated as candidate predictors. None met the retention criteria; consequently, WC was the sole predictor retained in each final model. The models presented in this table are therefore univariable and should not be interpreted as adjusted models. B represents the unstandardized regression coefficient, β the standardized regression coefficient, and the 95% CI refers to B. 6MWT, six-minute walk test; 30sCST, 30-second chair stand test; WC, waist circumference; SE, standard error; CI, confidence interval; VAS, visual analogue scale; KL, Kellgren–Lawrence

In post hoc sensitivity analyses, WC, pain intensity, and radiographic severity were entered simultaneously into multivariable regression models. Adjustment for pain intensity and KL grade produced only minimal changes in the magnitude of the WC coefficients compared with the primary univariable models (Table 3). WC remained associated with both functional outcomes, whereas pain intensity and KL grade were not independently associated with either outcome. Thus, the associations of WC with 6MWT and 30sCST performance were not materially altered by exploratory adjustment for pain intensity and radiographic severity.

**Table 3:** Forced-entry multivariable regression models for functional performance outcomes

Outcome	Model Fit	Predictor	B	SE	β	95% CI		*P*
6MWT	R² = 0.53 Adj. R² = 0.45	WC	−5.20	1.27	−0.71	−7.87 to −2.52		< 0.001
		VAS	−7.62	6.47	−0.004	−13.72 to 13.42		0.98
		KL	−0.15	25.85	−0.06	−61.94 to 46.70		0.77
30sCST	R² = 0.30 Adj. R² = 0.19	WC	−0.20	0.08	−0.51	−0.36 to −0.03		0.02
		VAS	−0.04	0.33	−0.03	−0.73 to 0.65		0.89
		KL	−0.64	1.60	−0.09	−3.98 to 2.70		0.69

Waist circumference, VAS pain score, and radiographic severity assessed using Kellgren–Lawrence grade were entered simultaneously into each model, irrespective of statistical significance. These models were used to examine whether the associations between waist circumference and functional performance were materially altered after adjustment for pain intensity and radiographic severity. B represents the unstandardized regression coefficient, β the standardized regression coefficient, SE standard erro, and the 95% CI refers to B

## Discussion

In this cross-sectional study of women with symptomatic, radiographic knee OA (KL II–IV), greater WC was associated with poorer performance in both the 6-minute walk test and the 30-second chair stand test. In contrast, calf circumference showed inverse associations with functional outcomes in unadjusted analyses; however, these associations were no longer evident after accounting for muscle quality. Together, these findings suggest that WC may provide a clinically useful proxy of adiposity-related functional vulnerability in women with knee OA, whereas CC should be interpreted with caution. Specifically, the negative association between calf circumference and functional outcomes appears to be driven by adiposity and impaired muscle quality rather than greater muscle mass per se, highlighting the importance of integrating assessments of muscle composition and function when examining sarcopenia-related impairments in this population.

Prior studies linking WC to mobility outcomes have often examined adults “at risk of OA” and focused on incident disability thresholds over time (e.g., failure to complete a standardized walk), rather than concurrent, performance-based capacity among symptomatic OA individuals [17, 28]. By restricting the sample to women with radiographic and symptomatic knee OA (KL II–IV) and using continuous, performance-based outcomes (6MWT, 30sCST), the present findings support WC as a correlate of current functional capacity even in the presence of structural knee OA.

Although WC is not a direct measure of visceral adiposity, it is widely used as a simple and clinically feasible anthropometric indicator of central adiposity and obesity-related metabolic risk [24, 29]. The association between WC and performance-based function observed in the present study is consistent with evidence linking central adiposity to systemic low-grade inflammation and metabolic dysregulation that may contribute to pain sensitivity, reduced activity tolerance, and broader functional limitation in OA [7, 30]. Given the exploratory and cross-sectional nature of the present study, larger longitudinal investigations are needed to confirm these associations and to determine the potential clinical utility of waist circumference for identifying functional risk and monitoring changes in physical performance over time. Furthermore, the integrated assessment of physical activity level, muscle strength, and comorbidities could help further clarify the underlying factors contributing to the association between WC and physical function in women with radiographic and symptomatic knee OA observed in this study.

Notably, radiographic severity and pain intensity were evaluated as potential covariates during exploratory model building but were not retained in the final models, whereas waist circumference remained significantly associated with functional performance, aligning with the recognized dissociation between structural OA severity and patient-relevant outcomes [31]. These findings combined with the post-hoc sensitivity analysis support the interpretation that systemic and metabolic factors may contribute to functional impairment beyond joint structural damage alone. Although pain is considered a clinically important determinant of functionality and quality of life in knee OA [31], pain intensity was not significantly associated with performance-based outcomes in the present sample, and VAS pain intensity was not retained as a significant predictor in the exploratory regression models. The sensitivity analysis showed that waist circumference remained associated with poorer performance on the 6MWT (B = − 5.20; 95% CI: −7.87 to − 2.52; *p* < 0.001) and 30sCST (B = − 0.20; 95% CI: −0.36 to − 0.03; *p* = 0.02), after adjustment for pain intensity and radiographic severity. Together, these findings suggest that waist circumference may be more associated with functional performance than pain intensity or radiographic severity in this sample, and that adiposity-related changes in lower-limb tissue composition, particularly impaired muscle quality, may contribute to this relationship. This interpretation is consistent with previous evidence indicating that functional limitations in OA are not fully explained by radiographic severity or pain symptoms, supporting the value of performance-based functional assessments in this population [22, 32].

Clinically, the high prevalence of increased or substantially increased metabolic risk based on WC in this sample reinforces the potential relevance of incorporating WC into routine assessment of women with knee OA as a simple indicator of metabolic vulnerability associated with poorer physical performance. In women, menopause-related shifts toward greater central adiposity may further exacerbate metabolic dysfunction and OA-related disability [33]. However, these findings should be interpreted cautiously, as other potentially relevant confounders, including physical activity level, muscle strength, medication use, menopausal status, and comorbidities, were not included in the analyses. Importantly, previous studies have also linked central adiposity and accumulation of metabolic risk factors to poorer physical function and greater walking difficulty in individuals with or at risk of knee OA [17, 34]. Together, these findings support the concept that waist circumference may reflect a broader clinical phenotype characterized by reduced mobility, lower activity levels, and functional vulnerability in knee OA populations [35].

Although calf circumference is frequently used as a pragmatic proxy for muscle mass in older adults [36–38], our findings indicate that in women with knee osteoarthritis, larger calf circumference does not necessarily correspond to better functional capacity. The inverse association observed in unadjusted analyses was partly sensitive to correction for subcutaneous fat thickness (attenuating the association in some comparisons) and was fully attenuated when models accounted for muscle quality. This suggests that CC in this clinical context likely reflects a mixture of tissues and processes, including peripheral adiposity and compromised muscle composition, rather than greater functional muscle mass.

This interpretation is consistent with reports of altered lower-limb muscle composition in knee OA, including increased intramuscular fat infiltration, fibrosis, and fatty degeneration within thigh musculature, all of which may increase limb girth without conferring functional benefit [39, 40]. From a screening perspective, our findings challenge the assumption that CC can be used as a straightforward indicator of sarcopenia-related functional risk in women with knee OA. Rather, they suggest that complementary assessments of muscle quality, such as ultrasound echo intensity and/or direct measures of performance or strength are necessary to better characterize functional risk in this population. This interpretation is also aligned with growing evidence from rheumatic and musculoskeletal diseases indicating that muscle quality and tissue composition may be more clinically relevant than muscle size alone, particularly in the context of sarcopenia and adiposity-related muscle alterations [41].

The relatively low calf subcutaneous fat thickness observed in the present sample should also be considered when interpreting these findings. Observed values ranged from 0.40 to 1.90 cm in the left leg and from 0.58 to 1.93 cm in the right leg, suggesting that local subcutaneous adiposity was not particularly high. Therefore, the absence of the expected positive association between calf circumference and functional performance, even under conditions of relatively low local adiposity, reinforces the need for caution when interpreting CC as a proxy for functional muscle mass. This limitation may be even more pronounced in populations with higher levels of adiposity.

A high prevalence of increased metabolic risk was observed in the present sample (77%) and WC assessment may help identify women with poorer physical performance and greater metabolic vulnerability who could benefit from targeted lifestyle and exercise interventions. BMI was evaluated as a candidate predictor during exploratory stepwise model building but was not retained in the final models. This finding indicates that BMI did not provide sufficient additional explanatory information under the adopted selection criteria; however, it should not be interpreted as demonstrating that WC contributes to functional performance independently of BMI. WC and BMI represent related but distinct anthropometric constructs. BMI reflects body mass relative to height, whereas WC is used as an indicator of central adiposity and obesity-related metabolic risk. Previous evidence suggests that WC may more closely reflect central adiposity and metabolic dysfunction than BMI alone, particularly in populations with age-related changes in body composition [29, 42]. Nevertheless, because BMI was not forced to remain concurrently with WC in the regression models, the contribution of WC beyond overall adiposity, as estimated by BMI, could not be determined. Larger studies with sufficient statistical power are needed to compare the relative and combined contributions of WC and BMI to functional performance in women with knee OA.

The findings of the present study should be interpreted considering several limitations. Firstly, the cross-sectional design precludes causal inference, and the relatively small sample size limits the precision, stability, and generalizability of exploratory multivariable analyses. Final sample size (*n* = 28) was slightly below the priori estimated target (*n* = 29); however, consistent associations between waist circumference and both functional outcomes were observed. Additionally, several factors that may influence functional performance in individuals with knee OA, including physical activity level, analgesic/NSAID use, comorbidities, menopausal status, and muscle strength, were not included in the exploratory regression analyses. Therefore, residual confounding cannot be excluded, and the observed associations should be interpreted with caution. Regarding calf circumference, some associations demonstrated wide confidence intervals and borderline statistical significance, indicating limited precision and the need for cautious interpretation. Secondly, our sample included only women, limiting generalizability to men with knee OA.

Thirdly, the 6-minute walk test was performed on a 7 m indoor flat course due to space constraints. Although the standard protocol recommends a 30 m corridor, shorter courses have been used when space is limited [43]. Because course length may influence the total distance walked, particularly through more frequent turns, results were interpreted as within-study functional performance measures and should not be directly compared with reference values or studies derived from standard 30 m protocols.

Unlike previous studies that primarily focused on OA risk or incident disability outcomes, the present study additionally explored the influence of muscle quality and tissue composition on anthropometric-functional relationships in women with symptomatic knee OA. Together, these findings suggest that waist circumference may represent an important anthropometric correlate of functional vulnerability, whereas radiographic severity and pain intensity were not retained as significant predictors in the exploratory regression models. These findings also highlight the importance of considering muscle quality when interpreting circumference-based anthropometric measures in this population. Given the exploratory and cross-sectional nature of the present study, larger longitudinal investigations are needed to confirm these associations and to determine the potential clinical utility of waist circumference as a simple screening marker for identifying women with knee OA who are at greater risk of reduced functional capacity and who may benefit from more detailed assessment of muscle function and physical performance, as well as targeted exercise or weight-management interventions.

## Conclusions

In women with symptomatic, radiographic knee osteoarthritis, greater WC was consistently associated with poorer functional performance and remained the only significant predictor in the final exploratory regression models. Calf circumference demonstrated inverse associations with functional outcomes in unadjusted analyses; however, these associations were attenuated after accounting for muscle quality, indicating that calf circumference alone may not be a reliable proxy for functional muscle mass in this population. Collectively, these findings indicate that waist circumference may represent a simple and clinically feasible anthropometric marker associated with functional vulnerability in women with knee osteoarthritis, while highlighting the importance of interpreting calf circumference in the context of adiposity and muscle composition.

## Supplementary Information

Below is the link to the electronic supplementary material.

Supplementary Material 1

## Declarations

### Generative AI and AI-assisted technologies in the writing process

During the preparation of this manuscript, the authors used OpenAI ChatGPT (GPT-4 and GPT-5) exclusively to assist with English language refinement, grammar correction, and improvement of text clarity. All AI-generated suggestions were critically reviewed, edited, and verified by the authors. The authors take full responsibility for the accuracy, integrity, and originality of the content presented in this manuscript.

### Disclaimer

This manuscript is an original work, has not been previously published, and is not under consideration for publication elsewhere. No part of the scientific content, analyses, results, or conclusions was generated by artificial intelligence. Any AI-assisted technologies used during manuscript preparation were limited to language editing and text refinement, and all outputs were critically reviewed and verified by the authors.
