# Supplementary material for: Central adiposity is associated with poorer functional capacity in women with knee osteoarthritis: a cross-sectional study with exploratory adjustment for pain intensity and radiographic severity
Source: Rheumatol Int. 2026 Jul 14;46(8):205. doi: 10.1007/s00296-026-06245-7 (PMC13364809; doi:10.1007/s00296-026-06245-7)
Supplement: Supplementary file 1 — Supplementary Material 1 [file 296_2026_6245_MOESM1_ESM.docx]

# STROBE Statement—Checklist of items that should be included in reports of cross-sectional studies

Manuscript Title: "**Central adiposity is associated with poorer functional capacity in women with knee osteoarthritis: a cross-sectional study with exploratory adjustment for pain intensity and radiographic severity."**Manuscript ID: RHEI-D-26-00823
Journal: Rheumatology International

| Item | Recommendation | Reported | Page No. |
| --- | --- | --- | --- |
| **Title and abstract** |  |  |  |
| 1(a) | Indicate the study’s design with a commonly used term in the title or the abstract | Yes | Abstract/methods |
| **1(b)** | Provide in the abstract an informative and balanced summary of what was done and what was found | Yes | Abstract |
| **Introduction** |  |  |  |
| Background/rationale 2 | Explain the scientific background and rationale for the investigation being reported | Yes | 1-2 |
| Objectives 3 | State specific objectives, including any prespecified hypotheses | Yes | 2 |
| **Methods** |  |  |  |
| Study design 4 | Present key elements of study design early in the paper | Yes | 2 |
| Setting 5 | Describe the setting, locations, and relevant dates, including periods of recruitment, exposure, follow-up, and data collection | Yes | 2-3 |
| Participants 6(a) | Give the eligibility criteria, and the sources and methods of selection of participants | Yes | 2-3 |
| Variables 7 | Clearly define all outcomes, exposures, predictors, potential confounders, and effect modifiers. Give diagnostic criteria, if applicable | Yes | 3-4 |
| Data sources/measurement 8* | For each variable of interest, give sources of data and details of methods of assessment (measurement). Describe comparability of assessment methods if there is more than one group | Yes | 3-4-5 |
| Bias 9 | Describe any efforts to address potential sources of bias | Yes | 3,8-9 |
| Study size 10 | Explain how the study size was arrived at | Yes | 2 |
| Quantitative variables 11 | Explain how quantitative variables were handled in the analyses. If applicable, describe which groupings were chosen and why | Yes | 4-5 |
| Statistical methods 12(a) | Describe all statistical methods, including those used to control for confounding | Yes | 4-5 |
| Statistical methods 12(b) | Describe any methods used to examine subgroups and interactions | Yes | N/A |
| Statistical methods 12(c) | Explain how missing data were addressed | Yes | 4-5 |
| Statistical methods 12(d) | If applicable, describe analytical methods taking account of sampling strategy | Yes | 4-5 |
| Statistical methods 12(e) | Describe any sensitivity analyses | No | N/A |
| **Results** |  |  |  |
| Participants 13(a) | Report numbers of individuals at each stage of study | Yes | 5 |
| Participants 13(b) | Give reasons for non-participation at each stage | No | N/A |
| Participants 13(c) | Consider use of a flow diagram | No | Not included |
| Descriptive data 14(a) | Give characteristics of study participants and information on exposures and potential confounders | Yes | 5 |
| Descriptive data 14(b) | Indicate number of participants with missing data for each variable of interest | No | N/A |
| Outcome data 15* | Report numbers of outcome events or summary measures | Yes | 5-6-7 |
| Main results 16(a) | Give unadjusted estimates and, if applicable, confounder-adjusted estimates and their precision | Yes | 5-6-7 |
| Main results 16(b) | Report category boundaries when continuous variables were categorized | Yes | 5-6-7 |
| Main results 16(c) | If relevant, consider translating estimates of relative risk into absolute risk for a meaningful time period | NO | N/A |
| Other analyses 17 | Report other analyses done—eg analyses of subgroups and interactions, and sensitivity analyses | Yes | 5-6-7 |
| **Discussion** |  |  |  |
| Key results 18 | Summarise key results with reference to study objectives | Yes | 7 |
| Limitations 19 | Discuss limitations of the study, taking into account sources of potential bias or imprecision. Discuss both direction and magnitude of any potential bias | Yes | 8-9-10 |
| Interpretation 20 | Give a cautious overall interpretation of results considering objectives, limitations, multiplicity of analyses, results from similar studies, and other relevant evidence | Yes | 6-7-8-9 |
| Generalisability 21 | Discuss the generalisability (external validity) of the study results | Yes | 6-7-8-9 |
| **Other information** |  |  |  |
| Funding 22 | Give the source of funding and the role of the funders for the present study and, if applicable, for the original study on which the present article is based | Yes | 11 |
